# Cocoa, Chocolate, and Human Health

**DOI:** 10.3390/nu12030698

**Published:** 2020-03-05

**Authors:** Benno F. Zimmermann, Sabine Ellinger

**Affiliations:** 1 Department of Nutritional and Food Sciences – Food Sciences, University of Bonn, Endenicher Allee 11-13, 53115 Bonn, Germany; benno.zimmermann@uni-bonn.de; 2 Faculty of Nutrition, Food, and Hospitality Sciences, Niederrhein, University of Applied Sciences, Rheydter Str. 277, 41065 Mönchengladbach, Germany

Cocoa has been used as a ceremonial and hedonistic food for thousands of years in the tropical parts of America and for hundreds of years in the western world. In the last decades, health-related aspects of cocoa have come into the focus of research. This Special Issue entitled “Cocoa, Chocolate, and Human Health” presents the most recent findings on cocoa and health in 14 peer-reviewed articles, including nine original contributions and five reviews from cocoa experts around the world.

Polyphenols of all kinds have attracted great attention because of their possible beneficial effects [1,2], but one has to keep in mind the enormous variety within the group of polyphenolic compounds. Cocoa and cocoa-containing food such as chocolate are particularly rich in flavan-3-ols, i.e., mainly epicatechin and its close relatives, the proanthocyanidins [3]. Bioavailability and metabolism of the native flavanols and the process-derived flavanols in cocoa and cocoa products are the subjects of three contributions. Stereoisomers of (–)-epicatechin that are generated during roasting and alkalization are less bioavailable than the native (–)-epicatechin [4]. Phase-II-conjugates of epicatechin and metabolites without an intact flavanol core like phenolic acids are found in plasma and urine [5]. Further metabolites such as valerolactones are formed by the gut microbiome by the degradation of non-absorbed flavanols. These microbial metabolites are present in human plasma at roughly five times higher concentrations than epicatechin conjugates [6]. The impact of all these metabolites for health has not yet been completely elucidated. Nevertheless, current data suggest possible evidence that these microbial metabolites might also be relevant for human health [7].

Besides flavanols, theobromine and other methylxanthines, peptides [8], and volatile aroma compounds [9] might also affect human health, e.g., theobromine, which seems to improve memory in rats [10]. Many studies, being intervention studies or epidemiological observations, do not focus on single compounds, but on cocoa as such; in some cases, enriched in polyphenols. In these studies, an observed effect can hardly be ascribed to a single constituent but proves the effectiveness of cocoa as a functional food. 

In this Special Issue, a positive influence of cocoa on hearing problems, exercise performance, and metabolic syndrome is discussed with mixed results. Hearing loss was found to be inversely associated with chocolate consumption in a middle-aged subgroup, but tinnitus did not depend on chocolate consumption [11]. In a review of thirteen clinical trials with athletes, a reduction of exercise-induced oxidative stress was found. However, regarding exercise performance and recovery, inconsistent results in literature did not allow a clear conclusion to be drawn [12].

There is evidence that cocoa flavanols may modulate some risk factors related to the metabolic syndrome, such as hypertension and disorders in glucose and lipid metabolism [13,14]. Several cardiometabolic parameters in type 2 diabetics were not affected by a flavanol-rich cocoa powder as simultaneous treatment with potent pharmaceuticals such as oral antidiabetic and antihypertensive drugs might have exhausted the effect of cocoa [15]. Also, the cocoa bean shell as a by-product of cocoa production contains valuable phytochemicals and can be used as an ingredient for functional food [16].

Three chapters focus on technical processes affecting cocoa components. During ripening and post-harvest processing, such as fermentation, drying, and roasting of cocoa beans, chemical changes occur to a lesser or greater extent, which concern almost all compounds. This has been known for many years regarding the flavanols, but proteins [17] and volatile compounds [9] are also formed or decomposed. Chocolate, the most popular cocoa product, shows remarkable losses in polyphenols and vitamin E during 18 months of storage. This is accompanied by changes in sensory profiles, while the flavor still remains acceptable [18].

Food and food supplements containing cocoa, enriched cocoa, or cocoa extracts are available for the costumers. The hoped-for effects are up to now only partly covered by scientific evidence. However, we have to bear in mind that manufacturers of such products do not only want to make the world a better place, but they also have financial interests.

As guest editors, we would like to acknowledge all the authors for their valuable contributions and the reviewers for their constructive remarks. Special thanks to the publishing team of *Nutrients* at MDPI for their professional support in all aspects of this Special Issue.
